# The effects of laboratory housing and spatial enrichment on brain size and metabolic rate in the eastern mosquitofish, *Gambusia holbrooki*

**DOI:** 10.1242/bio.015024

**Published:** 2016-01-21

**Authors:** Mischa P. Turschwell, Craig R. White

**Affiliations:** School of Biological Sciences, The University of Queensland, Brisbane, Queensland 4072, Australia

**Keywords:** Brain size, Enrichment, Resting metabolic rate, Laboratory housing, Intra-specific

## Abstract

It has long been hypothesised that there is a functional correlation between brain size and metabolic rate in vertebrates. The present study tested this hypothesis in wild-caught adult mosquitofish *Gambusia holbrooki* by testing for an intra-specific association between resting metabolic rate (RMR) and brain size while controlling for variation in body size, and through the examination of the effects of spatial enrichment and laboratory housing on body mass-independent measures of brain size and RMR. Controlling for body mass, there was no relationship between brain size and RMR in wild-caught fish. Contrary to predictions, spatial enrichment caused a decrease in mass-independent brain size, highlighting phenotypic plasticity in the adult brain. As expected, after controlling for differences in body size, wild-caught fish had relatively larger brains than fish that had been maintained in the laboratory for a minimum of six weeks, but wild-caught fish also had significantly lower mass-independent RMR. This study demonstrates that an organisms' housing environment can cause significant plastic changes to fitness related traits including brain size and RMR. We therefore conclude that current standard laboratory housing conditions may cause captive animals to be non-representative of their wild counterparts, potentially undermining the transferability of previous laboratory-based studies of aquatic ectothermic vertebrates to wild populations.

## Abstract (precis)

**Summary:** The discovery of a difference in brain size between adult mosquitofish living in the wild, in spatially enriched tanks and in bare tanks has implications for the effect of environmental enrichment on transferability of data from laboratory animals to their wild counterparts.

## INTRODUCTION

Many studies have postulated a functional correlation between brain size and resting metabolic rate (RMR) (Martin, 1981; Armstrong, 1983; Hofman, 1983; McNab and Eisenberg, 1989; Isler and van Schaik, 2006; Weisbecker and Goswami, 2010; Isler, 2011). RMR is defined as the metabolic rate of an animal in an inactive, post-absorptive, non-reproductive state (Burton et al., 2011) and individual variation in RMR is thought to have significant evolutionary consequences (Konarzewski and Diamond, 1995; Speakman et al., 2004; Konarzewski and Książek, 2013; White and Kearney, 2013). Although the brain represents only 0.1-1% of total body weight in vertebrates (excluding primates), brain tissue is energetically expensive to maintain, and requires nearly an order of magnitude more energy per unit weight than several other somatic tissues such as the liver and kidneys during rest (Mink et al., 1981; Rolfe and Brown, 1997; Wang et al., 2001; Soengas and Aldegunde, 2002; Javed et al., 2010). Brain tissue therefore contributes significantly to the RMR of an organism and is responsible for around 2-8% of resting oxygen consumption (Mink et al., 1981; McNab and Eisenberg, 1989; Nilsson, 1996; van Ginneken et al., 1996; Soengas and Aldegunde, 2002; Javed et al., 2010; Müller et al., 2011). Given the significant contribution of the brain to RMR, it is reasonable to expect a linear increase in RMR with increasing brain size, such that species with relatively large brains for their body size have a higher RMR than those with relatively small brains. Such relationships have been explored in mammals on multiple occasions, with mixed results (Hofman, 1983; McNab and Eisenberg, 1989; Isler and van Schaik, 2006, 2009; Weisbecker and Goswami, 2010, 2011). For example, Isler and van Schaik (2006) found a significantly positive correlation between residual (mass-independent) brain mass and RMR in mammals. Similarly, Weisbecker and Goswami (2010) demonstrate that eutherian mammals exhibit a positive relationship between brain size and BMR, whereas marsupials fail to exhibit such a relationship. Few studies have tested for an association between brain size and metabolic rate in ectothermic groups (Isler and van Schaik, 2009), and the only study available for fish found a weakly positive correlation between brain size and RMR, but no statistical analysis was undertaken (Albert et al., 1997).

Intra-specific studies of the association between brain size and RMR among individuals are considerably less common than comparative studies. In humans, individuals with relatively large brains have relatively high RMRs (Wang et al., 2001; Javed et al., 2010; Müller et al., 2011), but, again, data for ectothermic species are scarce. This is surprising, because brain size in ectotherms often shows significant plasticity, thereby providing an opportunity for manipulative tests of the association between brain size and RMR. For example, recent studies have demonstrated that, for a given body size, brain size differs between wild-caught and laboratory-reared individuals for both guppies (*Poecilia reticulata*) (Burns et al., 2009) and threespine sticklebacks (*Gasterosteus aculeatus*) (Park et al., 2012). The rearing environment of an organism throughout ontogeny often influences the growth of an individual and its organ structure, as many morphological and physiological traits are phenotypically plastic (Bedi and Bhide, 1988; Jacobs, 1996; Cotman and Berchtold, 2002; Gonda et al., 2012; Park et al., 2012). Numerous studies have demonstrated that animals including rats (Bennett et al., 1964a), flies (Technau, 1984) and crickets (Scotto Lomassese et al., 2000) reared in enriched environments have larger brain sizes for their body size than those raised in poorer conditions. Studies on rats have also demonstrated that environmental enrichment can invoke plastic changes to the brain in adults (Bennett et al., 1964b; Riege, 1971; Cummins et al., 1973).

Whether the alteration of housing environment following maturation in the wild can invoke plastic changes in metabolically active organs such as the brain remains poorly investigated in ectotherms. Although a large body of evidence supports an effect of rearing environment on brain size, manipulative laboratory experiments have failed to confirm such trends in fish. Burns et al. (2009) and Kihslinger et al. (2006) demonstrated that, for a given body size, wild-caught fish had larger brains compared to laboratory-reared counterparts in guppies and salmon (*Oncorhynchus tshawytscha*), respectively, but when they then separated laboratory-reared fish into enriched and standard environmental treatments, no significant difference was found in brain size between the two groups.

The present study had three overarching aims. Firstly, we tested for a mass-independent association between brain size and RMR by comparing the brain sizes and RMRs of wild-caught adult mosquitofish (*Gambusia holbrooki*) while statistically accounting for the relationship between body size and both brain size and RMR. Secondly, we examined the effect of environmental enrichment on brain size in adult mosquitofish by housing fish in spatially enriched and standard tanks and tested for the effect of spatial enrichment on brain size and RMR. We then compared the brain sizes and RMRs of wild-caught and laboratory-housed adult fish, while accounting for variation in body size.

## RESULTS

### Experiment 1: The relationship between brain size and resting metabolic rate (RMR) in wild-caught fish

**Fig. 1.**
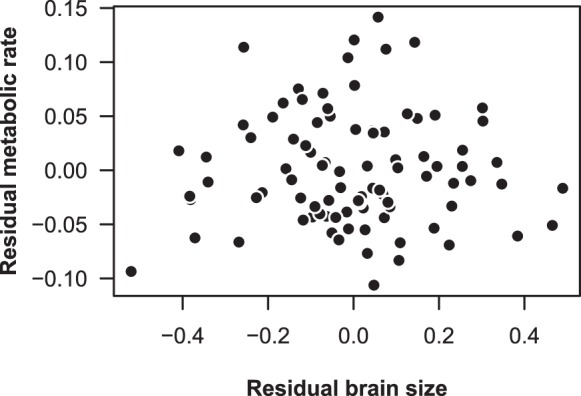
**The association between brain size and resting metabolic rate in wild *G. holbrooki*.** Residual values are from regressions on log body length and log body mass respectively, and the relationship is not significant (*n*=36 males and 55 females, *P*=0.968).

Accounting for the significant effect of body mass on RMR (*P*=0.005, Table 1), there was no relationship between brain size and RMR in wild-caught *G. holbrooki* (*P*=0.97, Table 1, Fig. 1). Principal Components Analysis showed that each individual brain region (Left optic tectum, right optic tectum, left telencephalic lobe and right telencephalic lobe) loaded positively onto component 1. The left optic tectum, right optic tectum and right telencephalic lobe all loaded positively onto component 2, while the left telencephalic lobe loaded negatively. There was no significant relationship between any of the calculated principal components (PC) of brain size and RMR (PC 1, *P*=0.97; PC 2, *P*=0.80; PC 3, *P*=0.99).

**Table 1.**
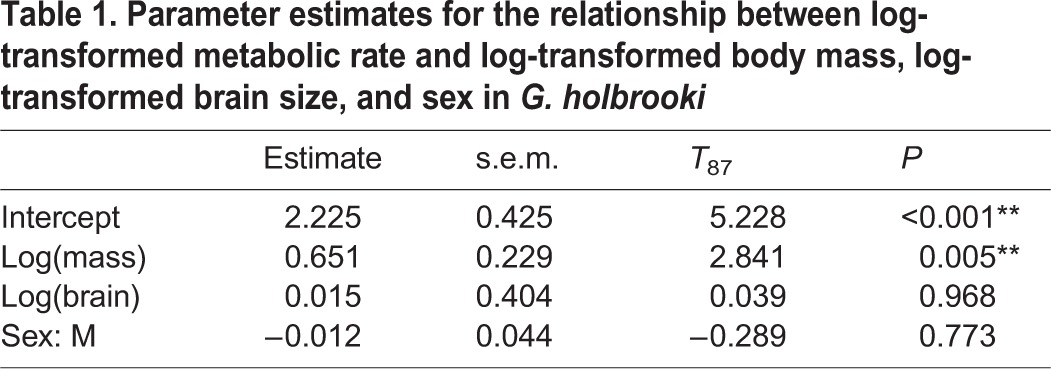
Parameter estimates for the relationship between log-transformed metabolic rate and log-transformed body mass, log-transformed brain size, and sex in *G. holbrooki*

### Experiment 2: The effect of spatial enrichment on brain size and RMR

#### Brain size

**Fig. 2.**
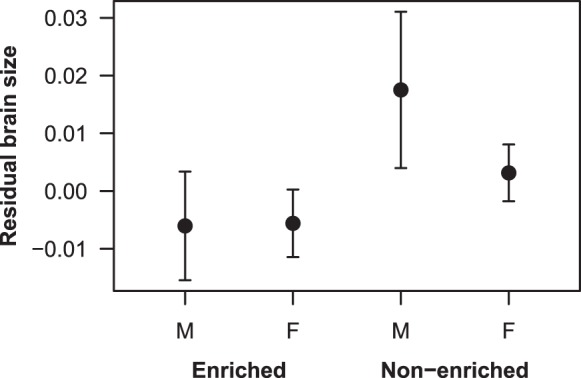
**The effect of spatial enrichment on brain size.** Individuals housed under standard non-enriched (S) conditions had significantly larger brains than individuals housed in spatially enriched (E) environments (*P*=0.046). Residual values are from regressions of log brain size on log body length. Plotted values are mean±s.e.m. *n*=31 (EM), 18 (EF), 34 (SM), 10 (SF). M, male; F, female.

Accounting for the significant relationship between brain size and body length (*P*<0.001; Table 2), spatial enrichment had a negative effect on brain size, with fish maintained in spatially enriched environments having significantly smaller brains than fish housed in bare (standard) tanks (*P*=0.046; Table 2, Fig. 2). Body condition was significantly positively associated with brain size (*P*=0.002), and males had significantly larger brains than females (*P*=0.04; Table 2, Fig. 2).

**Table 2.**
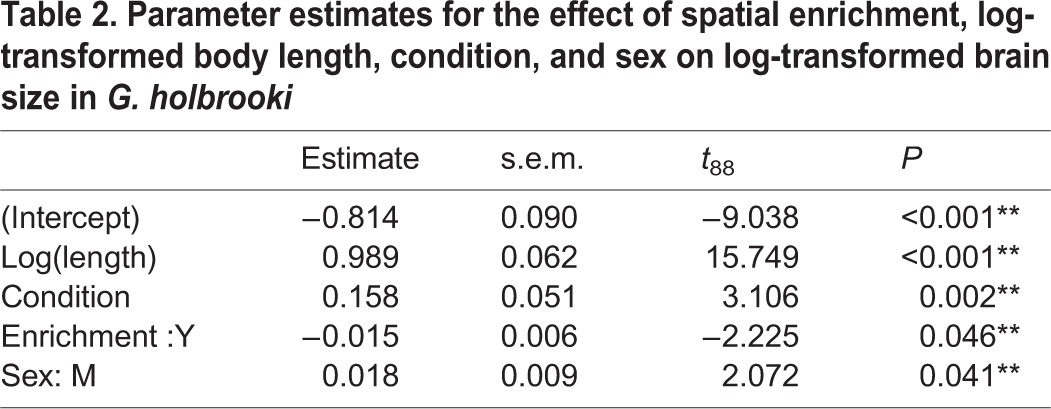
Parameter estimates for the effect of spatial enrichment, log-transformed body length, condition, and sex on log-transformed brain size in *G. holbrooki*

#### Metabolic rate

Accounting for the significant relationship between RMR and body mass (*P*<0.001; Table 2), housing treatment had no significant effect on RMR (*P*=0.796; Table 2), and there was no difference in RMR between sexes once variation in mass was included as a covariate in the multiple regression analysis (*P*=0.876; Table 2).

### Experiment 3: A comparison of wild-caught and laboratory-housed fish

#### Brain size

**Fig. 3.**
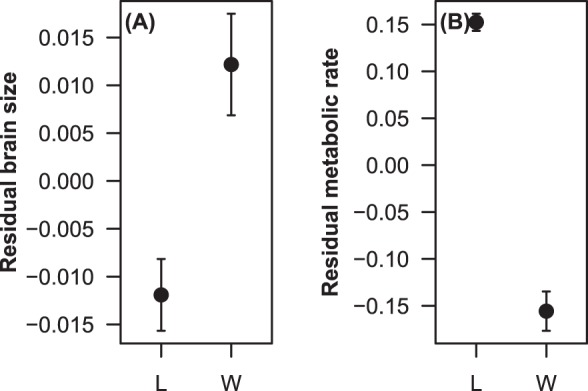
**The effect of laboratory housing on brain size and metabolic rate.** (A) The effect of laboratory housing on brain size in *G. holbrooki*. Wild-caught (W) individuals have significantly larger brains than laboratory-housed (L) individuals (*P*<0.001). Residual values are from regressions of log brain size on log body length. Values are mean±s.e.m. *n*=184 (64 M, 120 F). (B) The effect of laboratory housing on resting metabolic rate (RMR) in *G. holbrooki*. Wild-caught individuals have significantly lower resting metabolic rates than laboratory-housed individuals (*P*<0.001). Residual values are from regressions of log metabolic rate on log body mass and are mean±s.e.m. *n*=184 (64 M, 120 F).

Accounting for the significant relationship between brain size and body length (*P*<0.001; Table 3), the total brain sizes of wild-caught fish that had been maintained under laboratory conditions for less than two weeks were significantly larger than those of fish that had been maintained for more than six weeks (*P*<0.001; Table 3, Fig. 3A).

**Table 3.**
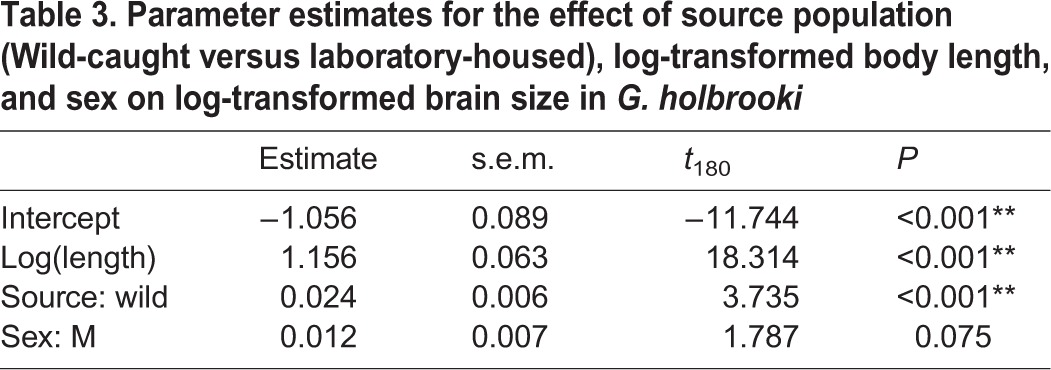
Parameter estimates for the effect of source population (Wild-caught versus laboratory-housed), log-transformed body length, and sex on log-transformed brain size in *G. holbrooki*

#### Metabolic rate

Accounting for the significant relationship between RMR and body mass (*P*<0.001; Table 4), wild-caught fish had significantly lower RMRs than fish housed in a laboratory environment (*P*<0.001; Table 4, Fig. 3B).

**Table 4.**
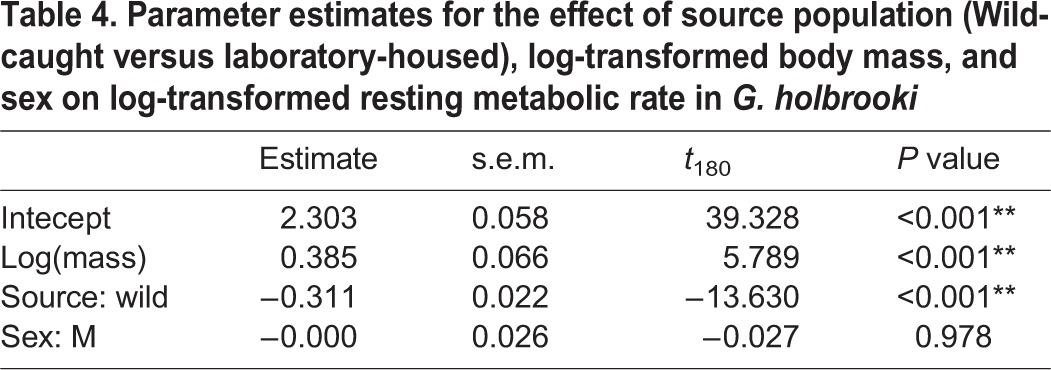
Parameter estimates for the effect of source population (Wild-caught versus laboratory-housed), log-transformed body mass, and sex on log-transformed resting metabolic rate in *G. holbrooki*

## DISCUSSION

The present study aimed to examine the relationship between brain size and RMR in wild-caught *G. holbrooki*, and the effects of environmental enrichment and laboratory housing on brain size and RMR. Contrary to predictions, there was no correlation between brain size and RMR in wild-caught fish. Enrichment had a negative effect on brain size, and males were found to have larger brains than females across all treatment groups. As expected, wild-caught fish had larger brains than laboratory-housed fish, though interestingly, laboratory-housed fish had significantly higher RMRs than wild-caught fish.

### Experiment 1: Investigating the relationship between brain size and resting metabolic rate in wild-caught fish

Brain tissue is energetically expensive to maintain (Mink et al., 1981) and an inter-specific, positive relationship between mass-independent brain size and RMR has previously been demonstrated on numerous occasions (Isler and van Schaik, 2006; Javed et al., 2010; Weisbecker and Goswami, 2010; Müller et al., 2011). The present study is the first, to our knowledge, to investigate the intra-specific relationship between brain size and RMR in an aquatic ectothermic vertebrate. Accounting for variation in body size, we found no correlation between brain size, or any brain region, with RMR in *G. holbrooki* (Fig. 1). These findings contrast results from a recent study of humans that found a positive association between brain size and RMR (Müller et al., 2011). The lack of an intra-specific association between brain size and RMR in *G. holbrooki*, compared to the positive association found for humans (Müller et al., 2011), might arise simply because the relatively large brain of humans contributes more to RMR than the smaller brains of *G. holbrooki*, but data for additional species will be necessary before it is possible to comment further on the generality of the intra-specific association, or lack thereof, between brain size and RMR in animals.

### Experiment 2: The effect of spatial enrichment on brain size and RMR

Contrary to the prediction that spatial enrichment would result in an increase in brain size, it was discovered that fish housed under spatially-enriched conditions for a minimum of six weeks had significantly smaller brains than those housed under standard conditions (Fig. 2). This result was unexpected, as numerous studies across multiple species have demonstrated that individuals exposed to enriched environments have significantly larger brains than their standardly housed counterparts (Bennett et al., 1964a, 1969; Technau, 1984; Scotto Lomassese et al., 2000). However, Welch et al. (1974) found that group living without an especially complex or enriched environment caused a significant increase in the mass of the cerebral cortex, telencephalon, and whole brain of rats. We therefore hypothesise that *G. holbrooki* living under enriched conditions were more able to isolate themselves from social interactions by hiding amongst the structure, thereby reducing the frequency of social interactions that may contribute to brain growth and plasticity. Similarly, socially isolated *Drosophila melanogaster* develop significantly smaller mushroom bodies than flies maintained in social groups (Technau, 1984). Rosenzweig et al. (1978) have also suggested social grouping as a potential contributor to cerebral changes and overall increases in brain size, though studies on rats suggest that social grouping alone cannot account for cerebral effects of enriched environments, and that changes are due to a combination of both the social environment and enrichment aspects.

An interesting result of the present study was the observation that males had significantly larger brains than females in both treatment groups (Fig. 2). The most likely explanation for this difference lies within the coercive reproductive strategy of *G. holbrooki*. Female mosquitofish almost never copulate willingly, and successful copulations are usually forced (Bisazza et al., 2001; Carter and Wilson, 2006). Termed ‘sneaky sex’, males stealthily approach females and rapidly insert their gonopodium to release sperm. The success of this reproductive strategy is relatively low, though males are highly active and will repeat the act numerous times to maximise the probability of fertilisation (Bisazza et al., 2001). More specifically, this reproductive strategy requires a significant amount of learning by males to successfully achieve copulation (Olsson and Shine, 1996). Learning has been linked with significant increases in brain size via the learning and memory hypothesis (Rosenzweig and Bennett, 1996). The learning and memory hypothesis suggests that increased stimulation from aspects including the social environment causes an increase in electrocortical activity, which leads to an increase in the metabolism of both neurons and glial cells. It is believed that an increase in the metabolic rate of these cells results in a net synthesis of cellular components, leading to larger brain sizes. A possible evolutionary explanation for the larger brains seen in male *G. holbrooki* may be therefore be attributed to positive selection for brain size in this coercive mating system. Given that learning is linked with plastic increases in brain size, it is also likely that the continual exhibition of reproductive behaviours and learning are contributing factors to the larger brain sizes seen in males. Future studies may wish to further investigate the effects of both environmental enrichment and social housing in an attempt to confirm findings from the current study. Additionally, investigating whether males and females show differential capacities for learning may provide information to explain the observed differences in brain size between sexes.

### Experiment 3: A comparison of wild-caught and laboratory-housed individuals

Wild-caught *G. holbrooki* have significantly larger brains than their laboratory-housed counterparts (Fig. 3A), as has been previously shown for guppies (Burns et al., 2009) and sticklebacks (Park et al., 2012). Non-reproducible environmental factors, natural chemical cues, interspecific interactions, an ever-changing physical environment, the effects of malnutrition, and the presence of predators and predation cues are just a few of the many variables believed to contribute to the differences seen in brain size between wild and laboratory populations (Rosenzweig and Bennett, 1996; Kihslinger et al., 2006; Gonda et al., 2010, 2012; Giesing et al., 2011). Without being continuously challenged with novel and altering habitats or being exposed to the threat of predators, we suggest that laboratory-housed fish experienced an overall reduction in brain stimulation, which resulted in a subsequent decrease in brain size. The effects of laboratory housing observed in the present study contrast the results of Burns et al. (2009), who demonstrated that 10 months in captivity had minimal effect on the relative size of the telencephalic lobes of adult guppies, whilst the present study found that a mere six weeks in a laboratory setting was sufficient to cause significant changes in brain size in mosquitofish. These responses are clearly species-specific, as the observed magnitude of brain plasticity is significantly different, even between these two species of poecillid.

Laboratory-housed fish had significantly higher RMRs than wild-caught fish (Fig. 3B). This result is most likely due to the chronic stress of being housed in a laboratory environment. Olsson and Dahlborn (2002) suggest that restrictive conditions limit an organisms' ability to control their physical and social environment. This ability to control such conditions is an important factor in determining on organism's reaction to an external influence (Olsson and Dahlborn, 2002). A stress reaction often occurs in response to an antagonistic stimulus if the organism cannot control the environment. Conversely, there may be no physiological response to a similar stimulus if the organism is in its natural habitat where it can display a degree of control over the situation (Wiepkema and Koolhass, 1993). The effect of social stress has been examined by Sloman et al. (2000), who housed brown trout (*Salmo trutta*) in tanks of two, leading to the development of a behaviourally dominant and subordinate individual. They found that the social stress of being a subordinate individual had implications at a metabolic level, with subordinates having significantly increased metabolic rates compared to dominant individuals. Similarly, Barton and Schreck (1987) examined the effect of physical stress on juvenile steelhead trout (*Oncorhynchus mykiss*), and found that subjecting fish to physical stress resulted in an increased metabolic rate.

### Conclusion

The present study has provided empirical evidence to suggest that there is no relationship between brain size and RMR in *G. holbrooki*. Confirming findings from previous studies, we found that wild-caught fish had significantly larger brains than laboratory-housed fish, and suggest that brains remain highly plastic throughout the lifespan of these fish. The cognitive consequences of smaller brains in laboratory-housed aquatic ectothermic vertebrates are not yet known, but factors such as reduced brain size and dendritic branching observed in laboratory rodents reared in un-enriched environments are associated with impaired problem-solving ability (Rosenzweig and Bennett, 1996). Larger brain sizes have been suggested to increase an organisms' fitness through greater cognitive function and increased survival, while smaller brains are associated with lowered cognition (Deaner et al., 2007; Burns and Rodd, 2008; Sol et al., 2008; Crispo and Chapman, 2010). Social behaviors, species interactions, environmental heterogeneity and predation are common in natural populations but cannot be replicated in laboratory environments (Kimball and Simon, 1985; Carpenter, 1996). Laboratory-housed individuals may therefore not be representative of their wild counterparts. This potentially undermines the transferability of previous studies on ectothermic vertebrates to wild populations. Current standard laboratory housing conditions could therefore compromise the use of laboratory maintained organisms for research, especially in behavioural, reproductive and genetic fields.

## MATERIALS AND METHODS

### Study species

*G. holbrooki* (Poeciliidae) were caught from the University of Queensland lakes at the St Lucia campus between February and May 2012. All experimental procedures were approved by the University of Queensland Native and Exotic Wildlife and Marine Animals ethics committee (certificate SIB/190/10/ARC).

### Experiment 1 – The relationship between brain size and resting metabolic rate (RMR) in wild-caught fish

#### Metabolic rate

The resting metabolic rates (RMR) of wild-caught *G. holbrooki* (*n*=91) were measured within two weeks of collection using closed system respirometry following Vaca and White (2010). Fish were maintained in 100 ml aerated glass chambers within a holding tank. The chambers were lined with black plastic to minimise external disturbances. Fish were fasted and acclimated to the respirometry chambers in a 25°C water bath for a minimum of 12 h prior to measurement. For measurements, respirometry chambers were sealed and the oxygen saturation was measured using a fibre-optic oxygen sensor (Ocean Optics FOXY-R, Lastek, Adelaide, Australia) connected to a temperature-compensated oxygen meter (TauTheta MFPF-100-2, Lastek). The chamber remained sealed and the decline in oxygen saturation was continuously monitored and recorded for a period of three hours. The first 30 min of data were allocated as a ‘settling in’ period to encourage resting behavior (Vaca and White, 2010), and were thus omitted from metabolic rate measures.

Blank trials (i.e. without a fish in the respirometry chamber) were run daily to quantify the background microbial oxygen consumption of the water. Blank trials were randomly assigned to each fibre-optic oxygen sensor channel to control for channel differences and their running was randomised throughout the time of day. At a minimum, one blank trial was run each day and a mean of all the trials was used as the control value for RMR calculations, as it was found that variation in daily blank measurements did not correlate with variation in daily measures of RMR. Fibre-optic oxygen sensors were recalibrated weekly using air- and nitrogen saturated water, and the water in the holding tank was completely changed every four to seven days to reduce microbial build up in the water.

RMR was estimated as rate of oxygen consumption (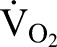, ml h^−1^), which was calculated from the time course of changes in oxygen concentration within the respirometry vials following Alton et al. (2007):
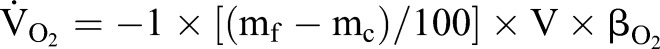


Where m_f_ is the slope of the fish, m_c_ is the control slope (used to account for microbial oxygen consumption in the water), V is the volume of the vessel minus the volume of the fish (ml) and 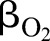 is the oxygen capacitance of water. The volume of individual fish was calculated from density (1.13 g ml^−1^), which was measured by weighing then placing five fish in a 10 ml graduated cylinder with a known volume of water and measuring the total displacement.

#### Brain size

Following measurement of RMR, fish were euthanised in a concentrated solution of clove oil. Total length was measured to 0.01 mm using digital calipers (Kincrome 200 mm No. 2351, Australia). Excess water on the body surface was removed using paper towel and fish were weighed to 0.1 mg using digital scales (MS204S, Mettler Toldeo, Switzerland). The brain case was then removed using a dissecting scalpel and 3 mm scissors, to expose the telencephalic lobes and the optic tecta, which was then photographed using a microscope-mounted digital camera (PIXELINK B686CF, Total Turnkey Solutions, Coburg, Australia) attached to a dissecting microscope (Olympus SZ61, Olympus Australia, Mount Waverley, Australia). The camera was linked to a PC running image-processing software (Pixelink Capture SE 3.1) and images were analysed using Pixelink software. A mesh grid of known length was placed next to the brains following dissections and was used to calibrate the imaging software. The area of each brain region (Left optic tectum, right optic tectum, left telencephalic lobe and right telencephalic lobe) as well as the total brain area was measured from dorsal images. As brain volume (calculated as depth×length×width) was found to be strongly correlated with the area calculated from dorsal photographs in the study by Burns et al. (2009), dorsal area was used as a surrogate for brain volume.

#### Statistical analysis

All statistical analyses were conducted using R version 2.15.1 (R Development Core Team, 2012), and α was set at 0.05 for all tests. Principal Components Analysis (PCA) was conducted using Ostats4 (http://www.maths.uq.edu.au/∼mrb/ostats/). Associations between brain size and RMR were examined using multiple regression with RMR as the dependent variable, and body length, brain size and sex as independent variables. Principal Components Analysis (PCA) was used to further investigate potential plastic changes in brain shape, rather than overall brain size. All data were log transformed prior to analyses.

### Experiment 2 – The effect of spatial enrichment on brain size and RMR

#### Spatial enrichment

To investigate whether plasticity in brain size is affected by housing environment, approximately 200 wild-caught *G. holbrooki* were randomly allocated to one of two laboratory-housing environments (enriched or standard). Each treatment environment was replicated 10 times, with treatments randomly assigned in a 20-tank recirculated water aquarium system, which ensured that there were no water quality differences among tanks and treatments (tank volume: 16.3 litre, total water volume of the aquarium system including filter sump: 390 litre). Reverse osmosis water treated with freshwater aquarium salt was used in the aquarium system. Appropriate denitrifying, chlorine and ammonia removal treatment was applied at fortnightly intervals (Prime, Seachem Laboratories, USA). Fish were maintained at 25±1°C in a temperature-controlled room for a minimum of six weeks prior to measurements, and a total of 93 randomly selected individuals were measured. Brain size and metabolic rate were measured as per experiment 1, with the exception that the measurement period for the decline in oxygen saturation was reduced to two hours as the decline in oxygen saturation for experiment 1 was found to be stable and consistent within this time frame.

Enriched environments were created according to recommendations made by Newberry (1995), who suggested the addition of tiers and vertical space to divide the tanks into different functional areas. Also, the addition of biologically relevant features such as mating rocks provides opportunities for exploration and is especially useful for animals adapted to unpredictable environments (Newberry, 1995). As such, spatially enriched tanks were enriched with three types of artificial aquarium plant. A small shrub, a medium-sized plant and a larger plant were used to provide numerous levels of spatial complexity. In addition, three pieces of poly-pipe were scattered throughout the tanks to provide additional three-dimensional structure. One was at ground level while two additional pieces were floating in the tank. Standard tanks were bare other than a layer of gravel.

#### Statistical analysis

Associations between brain size and housing environment were examined using multiple-regression with brain size as the dependent variable, and body length, housing environment and sex as independent variables. Similarly, the associations between RMR and housing environment were examined using multiple regression with RMR as the dependent variable, and body mass, housing environment (Enriched or Standard) and sex as the independent variables. All data were log transformed prior to regression analyses.

#### Condition factor

Brain size varies as a function of body size, though quantifying body size is difficult as it can be measured in multiple ways. Firstly as mass, which is a measure of body condition and structural size, or secondly as length, which is a solely a measure of structural size. These measures were not appropriate to include in multiple regression analyses as length and mass are highly correlated. Multicollinearity was avoided by calculating a condition factor, which described the relative mass of an individual for a given length. Heavier fish of a given length were said to be in better condition in comparison to lighter fish. Condition was calculated from the residuals of the regression of log mass and log length.

### Experiment 3 – A comparison of wild-caught and laboratory-housed individuals

The brain sizes and RMR's of wild-caught individuals were compared to laboratory-housed individuals. Laboratory-housed individuals included fish from both the enriched and standard groups, as the objective of this experiment was to test the overall effect of laboratory housing on brain size and RMR. Associations between brain size and source population (wild or lab) were examined using multiple-regression with brain size as the dependent variable, and body length, source and sex as independent variables. Associations between RMR and source were examined using multiple-regression with RMR as the dependent variable, and body mass, source and sex as independent variables. All data were log transformed prior to regression analyses.
